# Stoichiometry and structure of a lantibiotic maturation complex

**DOI:** 10.1038/srep42163

**Published:** 2017-02-07

**Authors:** Jens Reiners, André Abts, Rebecca Clemens, Sander H. J. Smits, Lutz Schmitt

**Affiliations:** 1 Institute of Biochemistry, Heinrich-Heine-University Duesseldorf, Universitaetsstraße 1, 40225 Duesseldorf, Germany

## Abstract

Lantibiotics are ribosomally synthesized antimicrobial peptides secreted by mainly Gram-positive bacteria. Class 1 lantibiotics mature via two modification steps introduced by a modification LanBC complex. For the lantibiotic nisin, the dehydratase NisB catalyzes the dehydration of serine and threonine residues in the so-called core peptide. Second, five (methyl)-lanthionine rings are introduced in a regio- and stereospecific manner by the cyclase NisC. Here, we characterized the assembly of the NisBC complex *in vitro,* which is only formed in the presence of the substrate. The complex is composed of a NisB dimer, a monomer of NisC and one prenisin molecule. Interestingly, the presence of the last lanthionine ring prevented complex formation. This stoichiometry was verified by small-angle X-ray scattering measurements, which revealed the first structural glimpse of a LanBC complex in solution.

Bacteriocins are a group of antimicrobial peptides produced by Gram-positive as well as Gram-negative bacteria and some of them undergo posttranslational modifications (PTM(s))^1^,^2^. Within bacteria they mostly remain inactive and are secreted via dedicated transport systems. So far, more than 750 different bacteriocins have been isolated from natural sources^1^ and the number constantly rises. Within the group of bacteriocins, a subfamily consists of ribosomally synthesized and posttranslational modified peptides, which are called lantibiotics. Here, non-natural amino acids and specific structures, which have an essential role on activity (e.g. lanthionine rings, dehydrated amino acids, heterocycles or head to tail cyclization of the peptide) are posttranslationally introduced. Common to all class I lantibiotics is a N-terminal leader sequence, which is crucial for the recognition by the PTM enzymes, secretion and for keeping the peptide in an inactive state within the cell. This leader sequence, which is often also called leader peptide, is fused to the so-called core peptide, in which all modifications occur.

Lanthipeptides contain the non-natural amino acids lanthionine or (methyl)-lanthionine and in case that they also display antimicrobial activity, these peptides are consequently called lantibiotics^3^,^4^. They are classified in four different classes (Type I-IV) depending on the enzymes involved in the PTM(s)^3^,^4^. Up to now, however, antimicrobial activities have only been reported for members of class I and II. Type I lantibiotics are modified by two different PTM enzymes, a lantibiotic dehydratase, LanB, and a lantibiotic cyclase, LanC. The enzyme LanB dehydrates specifically serine or threonine residues, whereas LanC catalyzes the thioether ring formation of the dehydrated amino acid and a C-terminally located cysteine residue within the core peptide via a Michael addition reaction^5^. This results in the formation of lanthionine (from Ser) or (methyl)-lanthionine (from Thr) rings, which are crucial for the activity as well as stability^6^,^7^,^8^,^9^,^10^,^11^,^12^. Type II lantibiotics are modified by a single enzyme called LanM, which catalyzes both, the dehydration and cyclization reaction, respectively^13^. In all cases the genes encoding the lantibiotic as well as the PTM enzymes are localized within in a single gene cluster and are valuable probes to identify lantibiotic operons in newly sequenced genomes^14^.

Nisin is a class I lantibiotic produced by several *Lactococcus lactis* (*L. lactis*) strains^15^,^16^,^17^. It contains characteristic dehydrated amino acids and five (methyl)-lanthionine rings, named rings A to E^18^,^19^. These rings are essential for the antimicrobial activity displayed against numerous Gram-positive bacteria^8^,^20^,^21^. The leader peptide as elaborated above is responsible for the recognition by the PTM enzymes, here called NisB and NisC, and furthermore is essential for the subsequent secretion by the ABC transporter, here called NisT^22^,^23^,^24^,^25^,^26^,^27^,^28^.

In detail, NisB dehydrates serine and threonine residues in the core peptide after ribosomal synthesis of nisin^29^,^30^,^31^,^32^. Seminal work on the mechanism of the dehydration reaction has demonstrated that a glutamate is transferred from glutamyl-tRNA^Glu^ to specific Ser/Thr side chains within the nisin core peptide introducing glutamylated intermediates^29^,^33^. After glutamate elimination, these Ser/Thr residues are converted to dehydroalanine and dehydrobutyrine with absolute stereoselectivity. Subsequently, NisC catalyzes a Michael addition of a C-terminal cysteine residue with the corresponding dehydrated amino acids to form thioether rings, the characteristic (methyl)-lanthionine rings^5^,^30^,^34^,^35^. The entire maturation process is schematically summarized in Supplementary Fig. 1. Although the activity of the single enzymes, NisB and NisC, has been demonstrated^29^,^33^,^34^,^35^, the assembly of a lantibiotic PTM complex has so far not been described *in vitro*.

In 1996, the first study revealed an interaction between NisC and NisB as well as between NisC and the ABC transporter NisT^36^. Furthermore, information about the directionality of the modification reaction was obtained suggesting a N- to C-terminal modification mechanism^9^. This is apparently in contrast to the PTM complex involved in NAI-107 maturation where an opposite directionality (C- to N-terminal) was discussed, which still is not fully understood^37^.

The PTM complex consisting of NisB-NisC-NisA was observed using a system that employed a His-tag fused to prenisin^38^. This allowed the isolation of the PTM complex from the cytosol of *L. lactis*. These associated proteins were identified as NisB and NisC, although in sub-stoichiometric amounts^38^.

Structural information is available for both PTM enzymes, NisB and NisC. However, this information is restricted to the isolated states of both enzymes. Two structures of lantibiotic dehydratases have been published^33^,^37^. The structure of NisB in complex with its substrate from *L. lactis*^33^ as well as the apo structure of MibB involved in NAI-107 biosynthesis from *Actinobacteria*^37^ were reported. Despite the low sequence homology (approximately 20%), the topology and fold of both proteins were very similar^37^. Interestingly, the amino acids involved in glutamylation and glutamate elimination are structurally highly conserved in both enzymes, which obviously suggests a fundamental similar mechanism of dehydration. The structure of NisC in the apo state has been solved with the catalytically important Zn^2+^ ion^34^,^35^.

Despite our increased knowledge of the nisin maturation reaction, little if any information about the complex stoichiometry of the PTM NisBC complex is available. Furthermore, the complex assembling process remains unclear. The nisin maturation machinery has been successfully explored to install PTMs in therapeutic peptides^39^,^40^,^41^. This substrate spectrum can be even further extended if the PTM complex could be employed *in vitro*.

Here, we describe for the first time an *in vitro* study revealing the formation of the nisin maturation complex composed of NisB, NisC and either unmodified or dehydrated prenisin peptide. Prenisin presents the essential trigger to initiate the *in vitro* formation of the maturation complex. Here, the -FNLD- box located within the leader sequence was identified as the crucial part in triggering complex assembly. Furthermore, our data demonstrated that the nisin PTM machinery consists of a functional dimer of NisB, a monomer of NisC and a single prenisin peptide. Once all rings are installed, as in fully modified prenisin peptide, the complex cannot assemble anymore suggesting a releasing factor upon formation of the last ring. Finally, structural information of the nisin modification complex was obtained by small-angle X-ray scattering (SAXS). Here, the same stoichiometry of the PTM complex was determined and revealed a tunnel located at the interface of NisB and NisC harboring the prenisin substrate. This result supports our *in vitro* studies and provides the first molecular picture of a class I lantibiotic maturation complex.

## Results

### Characterization of the modification enzymes NisB, NisC and the prenisin variants

Nisin contains several PTMs introduced by a proposed complex of NisB and NisC in an alternating manner^9^. To investigate the assembly of such a complex, we purified NisB and NisC to homogeneity. Previously, it was shown for NisC that the N-terminal His_6_-tag interfered with substrate binding and was therefore removed by thrombine cleavage prior to complex formation^22^. Both proteins were purified to a highly pure state as judged from SDS-PAGE analysis (Supplementary Fig. 2A and B). The oligomeric state of isolated NisB or NisC was analyzed via a combination of multi-angle light scattering and size exclusion chromatography (MALS-SEC)^22^,^25^. NisB is a dimer with a molecular weight of 237.5 ± 0.3 kDa (Supplementary Fig. 2C) and NisC is a monomer with a molecular weight of 48.1 ± 0.5 kDa. (Supplementary Fig. 2D) as previously reported^22^,^25^. The prenisin peptide and its variants were expressed and secreted by *L. lactis* NZ9000 and isolated via cation exchange chromatography as described^22^,^25^ (Supplementary Fig. 3).

Prior to the complex formation studies, the ability of the single proteins to bind prenisin was tested using MALS-SEC. Here, a dimer of NisB binds one unmodified prenisin molecule^25^ and the monomer of NisC binds also one unmodified prenisin molecule^22^ (Supplementary Fig. 4A and B) resulting in an increase of the observed molecular masses to 54.5 ± 0.6 kDa for NisC and 241.9 ± 0.4 kDa for NisB, respectively. Thus, purified NisB and NisC are capable to binding unmodified prenisin peptide. Higher amounts of prenisin did not result in higher molecular masses suggesting that both protein can only bind one prenisin molecule.

#### PTM complex assembly

**Figure 1:**
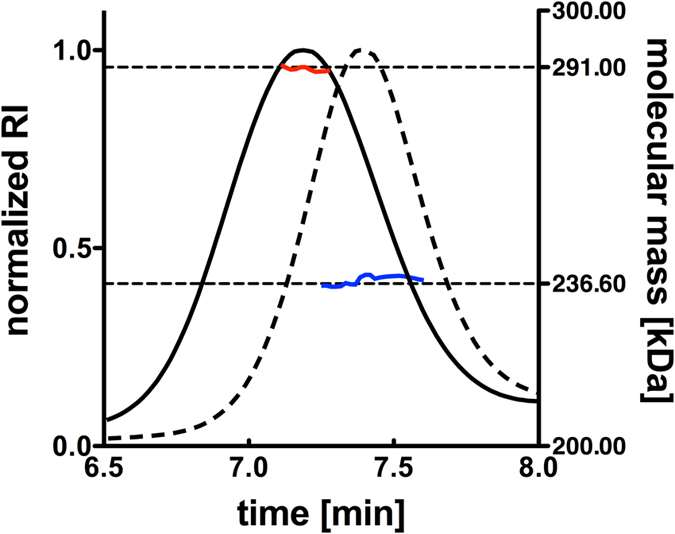
MALS-SEC analysis of the mixed protein samples consisting of NisB and NisC, in the presence or absence of the dehydrated prenisin peptide. The dashed black graph shows the elution profile of a mixture of 20 μM NisB and 160 μM NisC resulting in a molecular weight of 237.5 ± 0.3 kDa (blue line). The analysis of 20 μM NisB, 160 μM NisC and 200 μM dehydrated prenisin peptide is shown by the black graph, revealing an apparent molecular weight of 291.2 ± 0.9 kDa (red line) of the formed complex. The two black dotted lines indicate the theoretical molecular weight of an isolated NisB (236.6 kDa) dimer and of a complex consisting of a NisB dimer, a monomer of NisC and one prenisin peptide molecule (291 kDa).

**Figure 2:**
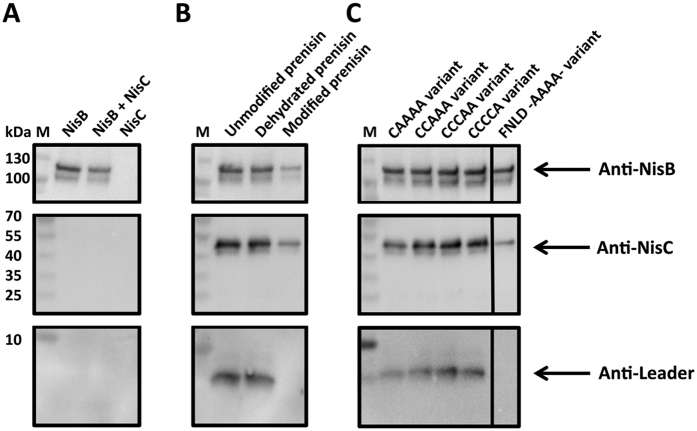
Western blot analysis of the PTM complex containing fraction. Elution fractions marked with a red box in the corresponding SDS-PAGE gels (Supplementary Figs 5, 7 and 8) were used for further analysis. The antibodies used are indicated with an arrow. (**A**) **M**: protein marker. **NisB**: The fraction resulting of the analytical SEC of 20 μM NisB. NisB and NisC: This sample represents the interaction analysis of 20 μM NisB and 160 μM NisC. **NisC**: The fraction resulting of the analytical SEC of 160 μM NisC. (**B**,**C**) In the following lanes the results of the complex formation for the different prenisin peptide variants are shown. PTM complex is formed and consists of NisB, NisC and prenisin peptide.

To assemble the nisin PTM complex *in vitro* we incubated NisB and NisC in different molar ratios ranging from 1:1 to 1:8 for 1 h at 25 °C and analyzed a potential complex formation by MALS-SEC. This resulted in two clearly separated elution peaks occurring at 7.4 min and 9.0 min. The first peak contained dimeric NisB with a theoretical molecular mass of 236.6 kDa (Fig. 1, black dashed line) and the second peak contained only NisC with a theoretical molecular weight of 48.5 kDa (not shown). To analyze this further we subjected single elution fractions of the SEC experiment to SDS-PAGE analysis (Supplementary Fig. 5) combined with Western blotting (Fig. 2A) using polyclonal antibodies against NisB and NisC, respectively. This revealed that NisB, even in the presence of a high excess of NisC, formed no complex with NisC (Fig. 1, black dashed line, Fig. 2A).

Next PTM complex formation was analyzed under conditions, in which either of the two enzymes, NisB or NisC, was preloaded with unmodified prenisin peptide. Upon saturation of NisB with unmodified prenisin peptide and subsequent incubation with 20 μM NisC prior to complex formation, a molecular mass of 263.8 ± 0.3 kDa was observed. This indicated formation of a NisB-NisC-NisA complex. When NisC was first saturated with unmodified prenisin peptide and subsequently incubated with 20 μM NisB, a molecular weight of 247.1 ± 0.4 kDa was observed for NisB (Supplementary Fig. 6).

This demonstrates that the presence of unmodified prenisin peptide triggers complex formation. However, the molecular mass determined in both experiments did not fit to any theoretically combination of the three components (1: 1: 1 or 2: 1: 1 or 2: 2: 2 or any other ratio) (Supplementary Table 2). This suggested that a fully assembled NisB-NisC-NisA complex with equimolar concentrations of NisB and NisC and an excess of unmodified prenisin peptide was not obtained under these experimental conditions.

**Figure 3:**
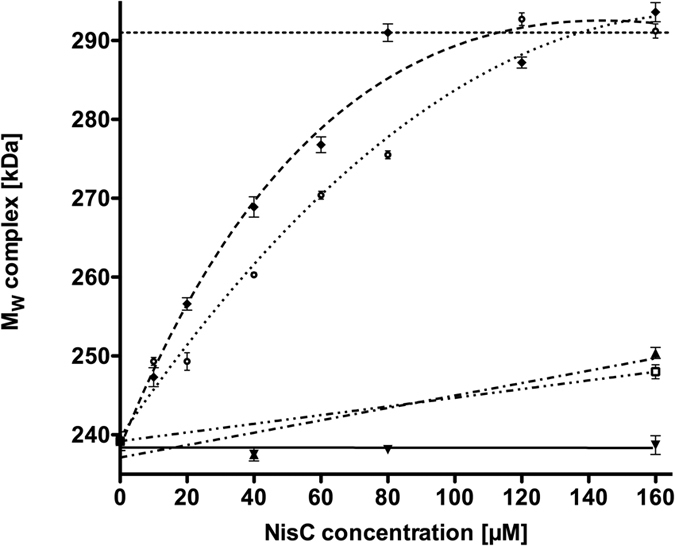
Assembly of the nisin maturation complex visualized via MALS-SEC. The molecular weight of the protein within the elution fraction was determined using MALS-SEC. The concentration of NisB (20 μM) and the different prenisin peptide variants (200 μM) were kept constant and the NisC concentration was varied (indicated on the X-axis). The upper dotted line shows the molecular weight of the theoretical PTM complex of 291 kDa. The molecular weight of NisB incubated with NisC is shown in ▾. With ♦, the molecular weight dependency of the complex with the unmodified prenisin peptide is shown. The dehydrated prenisin peptide profile corresponds to **⚪**. The molecular weight of the complex in the presence of the modified prenisin peptide is indicated by ▴. **◽** represents the dependency of the molecular weight of the complex in the presence of the -FNLD- box (-AAAA-) variant.

To obtain a fully assembled complex, we kept the concentration of NisB constant at 20 μM as well as the 10 fold-excess of unmodified prenisin peptide (200 μM). We increased the concentration of NisC stepwise from 10 μM to 160 μM and analyzed the molecular weights of the formed complexes via MALS-SEC. Here, we observed a gradual increase of the molecular weight at NisC concentrations of up to 80 μM (Fig. 3 and Supplementary Table 1). Further increase of the NisC concentrations to 160 μM did not change the observed molecular weight of 293.6 ± 1.2 kDa any further (Fig. 3, Table 1 and for a detailed view Supplementary Table 1). This suggested the presence of a stable complex of NisC/NisB/unmodified prenisin peptide at NisC concentrations of 80 μM or higher. The corresponding SDS-PAGE and Western blot analysis demonstrated the presence of both NisB and NisC in the elution fraction, together with the unmodified prenisin peptide (Fig. 2B and Supplementary Fig. 7A). After calculation of all possible stoichiometry’s, the nisin PTM machinery possessed a stoichiometry of 2:1:1 (see Supplementary Table 2).

#### The modification state of nisin dictates complex formation

The unmodified prenisin peptide initiated complex formation between NisB and NisC (Figs 2 and 3). This raised the question whether the modification state within the prenisin core peptide modulates complex formation? We therefore repeated the experiment employing dehydrated or fully modified prenisin peptide. In the latter case, all (methyl)-lanthionine rings are present, while in the dehydrated prenisin core peptide all serine residues and threonine residues are dehydrated^31^, but due to the lack of NisC during expression of prenisin, no (methyl)-lanthionine rings were introduced. We incubated the dehydrated and the fully modified prenisin peptide (200 μM) with 20 μM NisB and up to 160 µM NisC. We observed a complex of the PTM machinery after incubation of NisB and NisC with dehydrated prenisin peptide resulting in a molecular weight of 291.2 ± 0.9 kDa employing MALS-SEC analysis (Fig. 1 black line; Figs 2B and 3). This mass is identical within experimental error with the molecular weight determined for the unmodified prenisin peptide PTM enzyme complex. Importantly, an increase of the NisC concentration above 120 μM did not result in higher molecular weights indicating that no higher molecular weight PTM complex was formed (Figs 2B and 3, Table 1 and Supplementary Table 1). The corresponding SDS-PAGE analysis revealed the presence of NisB, NisC and dehydrated prenisin peptide in the elution fraction of the PTM enzyme complex (Supplementary Fig. 7B).

Incubating the fully modified prenisin peptide with NisB and NisC, resulted only in a small shift in the elution profile indicating that the modified prenisin peptide did not trigger complex formation (Figs 2B and 3, Table 1 and Supplementary Table 1 and Supplementary Fig. 7C). In MALS-SEC, a molecular weight of 250.4 ± 0.7 kDa was determined, which supported the idea of a weak interaction and reflects the lack of a stable PTM complex. Altogether, this demonstrated that fully modified prenisin peptide is not capable of inducing the formation of the completely assembled PTM complex, while unmodified and dehydrated prenisin peptide can do.

### The role of the (methyl)-lanthionine rings in complex formation

Only the presence of unmodified and dehydrated prenisin peptide triggered complex formation (see above). To investigate whether one or more of the five rings inhibits complex formation, four prenisin peptide variants were produced only differing in the number of (methyl)-lanthionine rings within the core peptide. The native prenisin core peptide contains five cysteine residues giving rise to the five (methyl)-lanthionine rings A-E after modification (Supplementary Fig. 1). By exchanging these cysteine residues subsequently to alanine, prenisin peptide variants were created which vary in the number of (methyl)-lanthionine rings. The CAAAA variant contains only ring A, CCAAA rings A and B, CCCAA rings A-C and CCCCA rings A-D. Here the variants are expressed in the presence of NisB and NisC, which ensures that these variants are dehydrated and the lanthionine rings are present when a cysteine residue is still available. After purification, we incubated 20 μM NisB, 160 μM NisC and 200 μM of these different ring variants of the prenisin peptide for 1 h at 25 °C and analyzed the reaction mixtures by analytical SEC. The corresponding SDS-PAGE analysis is shown in Supplementary Fig. 8A–D. The analysis demonstrated that all ring deficient prenisin peptide variants are capable to form the PTM complex. In all cases, a co-elution of NisB, NisC and the prenisin peptide was observed. The co-elution fraction was furthermore analyzed by Western blot (Fig. 2C) to visualize the presence of NisB, NisC and the prenisin peptide variant used. This result demonstrates that the PTM complex is obtained, in the presence of at least one up to four (methyl)-lanthionine rings. Only when the last (methyl)-lanthionine ring (ring E) was present, i.e. fully modified prenisin peptide, no complex formation was detected. This suggests that coming to the last ring or dehydratable residue at the C-terminus might stimulate the dissociation of the entire PTM complex.

#### The recognition motive within the leader peptide: the -FNLD- box

We showed above that the modification state of the core peptide has a profound influence on complex formation. Next, we concentrated on the role of the N-terminal leader peptide in the assembly of the modification complex. The single NisB and NisC enzymes recognize the highly conserved -FNLD- box motif within the leader peptide^22^,^25^. MALS-SEC analysis using the -FNLD- to -AAAA- mutant resulted in a molecular weight of 248.0 ± 0.9 kDa, which indicated low efficiency of PTM complex formation (Figs 2C and 3, Table 1 and Supplementary Fig. 7D). The SDS-PAGE analysis of the analytical SEC revealed a minor shift of NisC and only low amounts of NisC co-eluted with NisB (Supplementary Fig. 7D). This suggests that the -FNLD- box has an important role during complex formation and demonstrated that both, the leader and the core peptide, are essential for the assembly of the nisin PTM complex. This is in line with the observations that this prenisin peptide variant is poorly modified *in vivo*^23^,^26^,^27^,^38^.

### Visualization of the nisin modification complex with small-angle x-ray scattering (SAXS)

**Figure 4:**
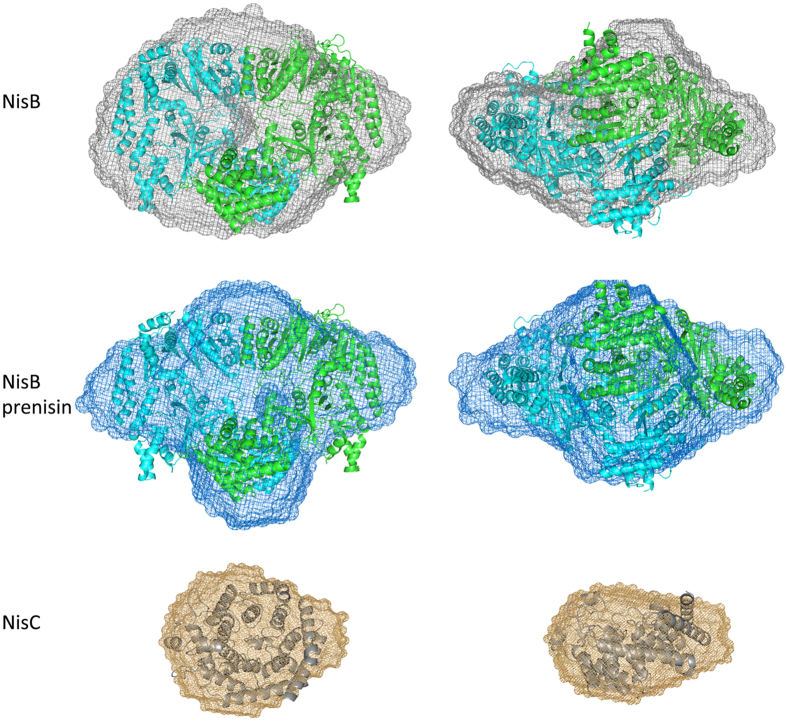
SAXS analysis of apo-NisB, NisB-prenisin and NisC. The volumetric envelopes are shown for NisB (grey mesh), NisB-prenisin (blue mesh) and NisC (orange mesh) as calculated from the scattering data using DAMMIF^43^. The structure of NisB (PDB code: 4WD9) and NisC (PDB code: 2GOD) were docked into the volumetric envelopes using SUPCOMB^44^.

We used SAXS to obtain a structural glimpse of the fully assembled PTM complex. Here, we applied 200 μl of each enzyme, NisB (200 μM) and NisC (470 μM), respectively, on a Superdex 200 column and measured the X-ray scatter (Supplementary Fig. 9). We estimated the molecular weight from the POROD volumes^42^ (Supplementary Table 3). This resulted in a molecular mass of 239.26 kDa for the NisB sample corresponding to a dimer, and in a molecular mass of 51.56 kDa for NisC, which corresponds to the monomeric state. These results fitted to the theoretically calculated masses deduced from the corresponding sequences (Supplementary Table 3) and were in-line with our MALS-SEC results (Table 1). Next we applied the same method to a NisB sample saturated with dehydrated prenisin peptide (200 μM NisB, 2 mM prenisin). Here, we incubated NisB with prenisin peptide, which should result in saturation of the enzyme. The calculated molecular weight of 256.54 kDa indicated that dehydrated prenisin peptide bound to NisB. The volumetric envelope was calculated from the background-subtracted scattering curves using DAMMIF^43^ (Fig. 4).

**Figure 5:**
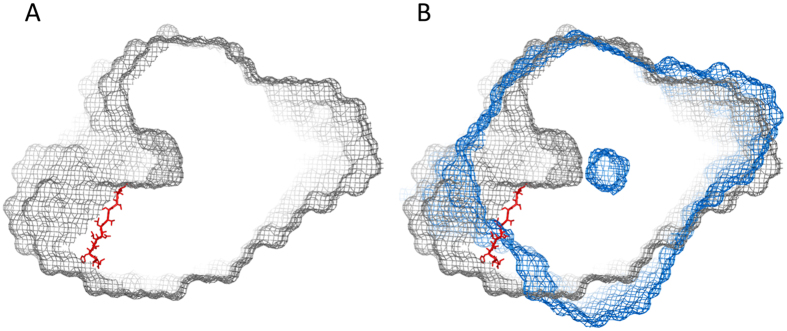
Comparison of the NisB and NisB-prenisin peptide volumetric envelopes as obtained by SAXS. (**A**) The volumetric envelope of NisB is shown as a grey mesh. A 30 Å clip was calculated around the leader peptide as derived from the fitted NisB structure (highlighted in red stick representation) (**B**) An overlay of the NisB envelope (grey mesh) and the NisB-dehydrated prenisin peptide envelope (blue mesh) highlighted that the observed tunnel is closed once dehydrated prenisin peptide is bound to NisB.

The structure of NisB (PDB code 4WD9) and NisC (PDB code 2GOD), which were solved previously by X-ray crystallography, were superimposed into the volumetric envelopes using the program SUPCOMB^44^ (Fig. 4), demonstrating that the envelope of the enzymes in solution nicely fitted to the published X-ray structures (Fig. 4). Interestingly in the map of the apo-NisB a tunnel on only one site of the envelope was observed (Fig. 5A), which might serve as an entrance to the binding site of prenisin. The leader sequence was visible in the X-ray structure of NisB and after fitting the model into the volumetric envelope obtained by SAXS, it was localized in close vicinity of the observed tunnel (highlighted in red in Fig. 5A). This further strengthens the suggestion that the tunnel might be the binding site for the prenisin molecule. This tunnel however appears to be closed in the volumetric envelope derived from our SAXS measurements of NisB saturated with dehydrated prenisin peptide. An overlay of both envelopes revealed that the tunnel is blocked in the NisB-dehydrated prenisin peptide map (Fig. 5B). Please note that the measurement of both samples were performed at similar concentrations and resulted in volumetric envelopes of comparable resolution. This suggests that the tunnel represents an opening for prenisin, which is blocked once prenisin is bound. Furthermore, this tunnel points into the active site of NisB and to the residues involved in the dehydration reaction as well as glutamylation^23^,^33^,^38^.

**Figure 6:**
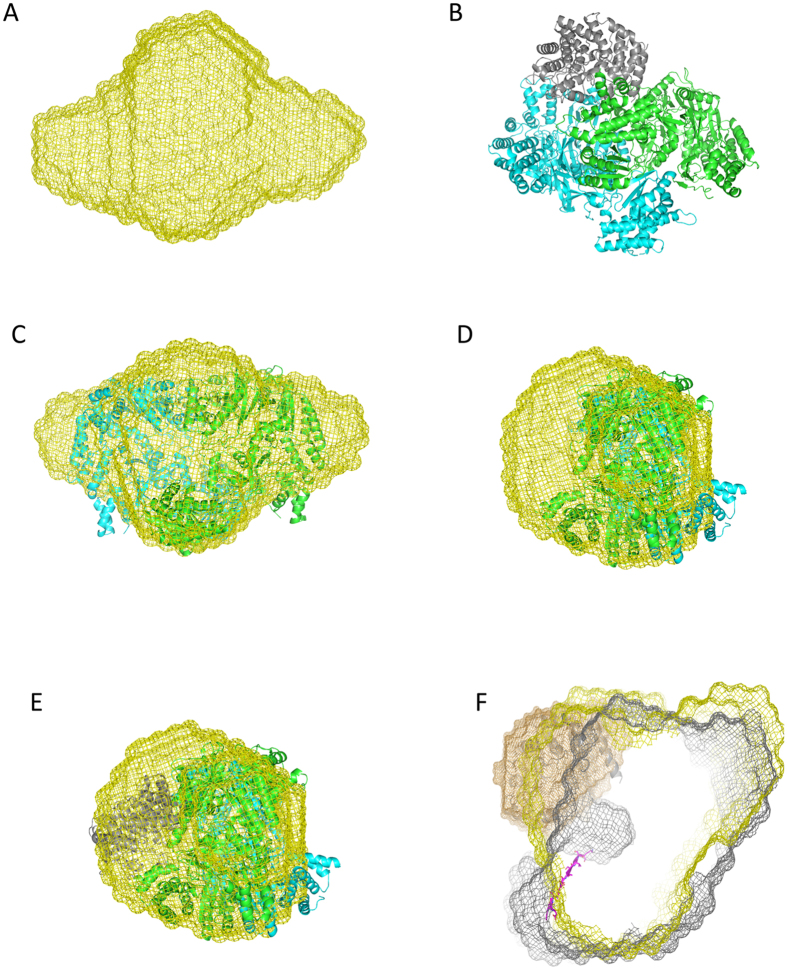
SAXS analysis of the fully assembled PTM complex. (**A**) The volumetric envelope of the fully assembled complex is shown as a yellow mesh. (**B**) The assembled PTM complex as deduced from the program SASREF^54^ (The monomers of NisB are colored in blue and green, NisC in dark orange). (**C**) Front view of the PTM complex with only NisB highlighted, (**D**) side view of the PTM complex with only NisB highlighted, (**E**) fully assembled PTM complex with the complex aligned to the volumetric envelope. (**F**) Zoom in into the PTM complex identical to Fig. 5. The observed tunnel in NisB is occupied by dehydrated prenisin peptide and also NisC is located at the same interface of the protein.

Next we also measured the fully assembled PTM complex. This revealed a molecular weight of 275.1 kDa as deduced from the POROD volume indicating that the PTM complex was not fully assembled, which is likely due to the dilution effect occurring during SEC. Even after increasing the concentration of the PTM complex, the amount of formed complex did not increase. The different molecular weights from the POROD Volume were also supported by the calculated Radius of Gyration (R_g_) as well as estimated D_*max*_. We could observe an increase for the PTM complex in comparison to isolated NisB (Supplementary Table 3). The overall shape of the obtained volumetric envelope revealed additional density on top of the NisB volume near the tunnel observed in the NisB experiment (Fig. 6A–D), suggesting that NisC is localized at that position. Furthermore, no additional volume large enough to accommodate another NisC molecule was observed. This demonstrates that the stoichiometry obtained by SAXS was identical to the stoichiometry deduced by SEC-MALS. Independently, we used the program SASREF to calculate the shape of the PTM complex using the structures of NisB and NisC and the experimental scattering data. Here a similar location of NisC was observed. NisC is again localized on top of the tunnel suggesting that this represents the stable conformation of the PTM complex *in vitro* (Fig. 6D–F). NisC has a bowl like structure and the active site points again towards the tunnel (Fig. 6F). This suggests that NisC is localized next to the prenisin binding site observed for NisB within the PTM complex.

## Discussion

Bacteriocins are peptides produced by bacteria and there is one specific class, called lanthipeptides, contains PTMs introduced by specific enzymes, which can be either a single protein (LanM) or two proteins (LanB and LanC, respectively)^3^,^13^,^16^,^17^. In the case of systems containing LanB and LanC, a complex of both enzymes is proposed to be the catalytically active species, but direct experimental evidence of its existence is rare. Here, we provide the first *in vitro* data on the assembly of the nisin PTM complex consisting of the dehydratase NisB and the cyclase NisC. The enzyme NisB and NisC are proposed to work in an alternating fashion to introduce the PTMs in the core section of the prenisin peptide^9^. We only observe the assembly of the PTM complex in the presence of unmodified and dehydrated prenisin peptide (Figs 2 and 3). This is in line with *in vivo* and *in vitro* data, in which direct interaction(s) between NisB and NisC was never observed without the presence of the substrate^3^,^17^,^36^,^38^,^45^. Purification of the prenisin peptide via an affinity tag directly from the cytosol of *L. lactis* resulted in elution fractions containing both, NisB as well as NisC^38^. This strongly suggested that a fully assembled maturation complex was present within the cytosol. The data presented here provides the first *in vitro* reconstitution of a lantibiotic PTM complex using the three separately purified components.

The stoichiometry of the PTM complex was determined to be 2:1:1 (NisB: NisC: prenisin peptide) by two independent approaches, SEC-MALS as well as SAXS.

The structure of NisB also revealed a dimeric organization with prenisin bound although electron density only for the region surrounding the -FNLD-box within the leader peptide was clearly visible^33^. Within this NisB structure the leader was detected in both monomers, which might be due to the simultaneous overexpression of both, NisB and prenisin, respectively, in combination with the lack of NisC. In the recently published structure of MibB, no substrate was observed^37^. While comparing the binding site of both enzymes, it became obvious that this particular region is rather flexible. This suggests that upon substrate binding NisB undergoes a conformational change as observed in the SAXS experiment. The differences are however subtle.

### Interaction between NisB, NisC and the prenisin core peptide

Here, we demonstrated that complex formation between NisB and NisC strictly relies on the presence of prenisin, i.e. either the unmodified or the dehydrated peptide variant. When the cyclization reactions are completed and five (methyl)-lanthionine rings are present in the prenisin peptide, only a very minor amount of PTM complex can be obtained. This might reflect the *in vivo* situation, in which fully modified prenisin peptide is released from the PTM complex as soon as the (methyl)-lanthionine rings are formed and handed over to the dedicated transport system NisT. Since all three components were apparently localized at the membrane of *L. lactis*^36^, it seems plausible that an even larger complex consisting of the PTM enzymes and the ABC transporter exists within the bacterial cell. Installing the lantibiotic PTMs and the subsequent secretion benefits from an efficient coupling of lantibiotic biosynthesis, maturation and secretion.

Within the leader peptide of class I lantibiotics, the -FNLD- box is highly conserved^26^ and was identified as the recognition motif for the isolated enzymes NisB and NisC, respectively^22^,^25^,^38^. With the exchange of the -FNLD- motif against four alanines (-AAAA-), the formation of the PTM complex was drastically reduced (Figs 2 and 3 and Table 1). This is in contrast to *in vitro* results with isolated proteins, where no binding was observed^22^,^25^. But we have to stress that in these *in vitro* studies only low concentrations of the -AAAA- mutant of prenisin peptide were used and that the concentrations used in this study were several times higher. Nevertheless, our result clearly support that this highly conserved motif possesses a strong effect on complex formation as exemplified by the -AAAA- mutant. This was previously also highlighted by *in vivo* studies, which demonstrated that this variant is poorly modified and contains almost no dehydrations or cyclisation within its core peptide^23^,^26^,^27^,^38^.

SAXS analysis revealed that in the apo-NisB structure a tunnel is present in close proximity of the binding site of the leader sequence. This tunnel is not observed within the NisB-dehydrated prenisin peptide complex. This suggested that the dehydrated prenisin peptide occupies this space. Interestingly, only one tunnel is observed indicating that one prenisin molecule can bind to NisB in solution. The other possible binding site appears to be closed. Furthermore, we observed that NisC is localized next to this tunnel with its active site pointing towards the prenisin binding site as well as the region of NisB, which contains the residues important for the dehydration and glutamylation reactions^29^,^33^.

Taken all data together the dehydration reaction within the core peptide likely changes the conformation of prenisin such that the active site of NisC becomes accessible, which is capable to cyclize the first ring. This would be inline with the model proposed that nisin is modified via a ping-pong mechanism^9^,^46^ were dehydration and cyclization occur in an alternate fashion. Due to (methyl)-lanthionine ring formation, the peptide shifts forward and the second dehydration step can occur. It remains unclear whether the position of the leader peptide is also shifted during this process and consequently moves out of the PTM complex. *In vivo* this might be favorable since the leader sequence needs to be recruited by the ABC transporter NisT. Finally, the presence of ring E prevented complex formation, or stimulate the dissociation. Apparently, the conformation of the core peptide is different in comparison to the conformation of nisin that contains rings A-D. This likely is ensured by the more bulky intertwined conformation of ring D and E^19^.

In summary, the data obtained in this study identified two factors influencing complex formation of the maturation enzymes NisB and NisC, respectively. First the core peptide, it can be dehydrated and also particular modified. *In vitro* the presence of the last (methyl)-lanthionine ring, ring E, abolished complex formation. Second, the N-terminal leader peptide plays an important role. The highly conserved -FNLD- box is an essential recognition factor for the modification enzymes NisB and NisC. Finally, the MALS-SEC analysis revealed the first quantitative data elucidating the stoichiometry of the nisin maturation complex. This complex revealed a molecular weight of approximately 291 kDa corresponding to a stoichiometry of 2:1:1 (NisB/NisC/prenisin peptide) *in vitro*.

## Materials and Methods

### Cloning the prenisin ring deficient variants

For producing ring deficient prenisin peptides, a shuttle vector accessible for the bacteria *L. lactis* and *E. coli* was created^47^. The correctness of the obtained plasmids was successfully verified by sequencing.

#### Purification of NisB, NisC and the prenisin peptide variants

NisB was expressed in *L. lactis* and purified as previously described^25^. The expression and purification of NisC was performed as described in ref. 22. Briefly, the dehydratase NisB was homologously expressed in *L. lactis* NZ9000 and purified to homogeneity via immobilized metal ion affinity chromatography (IMAC) followed by SEC. NisC was heterologously expressed in *E. coli* BL21 and isolated via a three-step purification strategy. The first step was again IMAC chromatography using a TALON^®^ Superflow Cartage Colum, followed by a SEC purification step. The N-terminal His_6_-tag of NisC was cleaved off by thrombin treatment. Non-digested NisC was removed via a second IMAC step.

The prenisin peptide purification was performed as described in ref. 22. For the determination of the prenisin concentration and the variants a RP-18 HPLC column was used^22^. In general prenisin is expressed via a two plasmid system. On the first plasmid wildtype prenisin or the cysteine variants of core nisin or the FNLD variants of the leader part of nisin is expressed. The second plasmid contains the PTM complex consisting of NisB, NisC and NisT. By varying the latter plasmid, the modification status of prenisin can be varied. Here, the deletion of NisC leads to a prenisin peptide variant, which is dehydrated but no lanthionine rings are installed. Similarly, the deletion of both, NisB and NisC, on the plasmid results in a prenisin peptide varians where no dehydration and no cyclization are present. The differences in modification are highlighted in Supplementary Figure S1.

#### Analysis of complex formation

To determine the molecular weight and stoichiometry of the NisB/NisC/prenisin peptide complex, a combination of multi-angle light scattering and size exclusion (MALS-SEC) was used to visualize complex formation. The analyses were performed on an Agilent 1260 HPLC System in combination with a triple-angle light scatter detector (miniDAWN TREOS) and a differential refractive index detector (Optilab rEX – both Wyatt Technology Europe).

Analysis of isolated NisB and NisC were performed by injection of 200 μL of a 20 μM solution of each protein. The second step was the analysis of prenisin peptide bound proteins. We used 20 μM NisC, respectively 20 μM NisB and incubate it with 200 μM prenisin peptide for 1 h at 25 °C.

For the initial complex analysis we saturated NisB with the unmodified prenisin peptide and analyzed the mixture by MALS-SEC. Here, we used 20 μM unmodified prenisin peptide saturated NisB and incubate it with 20 μM NisC for 1 h at 25 °C. The same analysis was performed with 20 μM unmodified prenisin peptide saturated NisC and subsequent incubation with 20 μM NisB. A Volume of 200 μL was applied on an Agilent BioSEC-5 colum (300 Å, 7.8 × 300 mm) pre-equilibrated with MALS buffer (50 mM HEPES-NaOH, pH 7.5, 500 mM NaCl) at a flow rate of 1.0 mL/min or on a Superdex 200 10/300 increase colum (GE Healthcare) at a flow rate of 0.75 mL/min. Data-analysis was performed with the ASTRA software package (Astra V 5.3.4.20) (Wyatt Technology).

To visualize complex formation, we kept the concentrations of NisB (20 μM) and of the prenisin peptide variants (200 μM) constant in the different samples. We used only different concentrations of NisC from 10 μM to 160 μM.

#### Analytical co-elution studies

The co-elution studies were performed on a Äkta Micro system using a Superdex 200 PC 3.2 column (GE Healthcare) equilibrated with 50 mM HEPES-NaOH, pH 7.5, 500 mM NaCl with a flow rate of 0.05 mL/min.

A 50 μL reaction mixture consisting of 20 μM NisB, 160 μM NisC and 200 μM prenisin peptide variant was incubated for 1 h at 25 °C and subsequently applied to SEC analysis. Elution was observed at 280 nm. After co-elution, the corresponding fractions were analyzed by a 4–20% gradient Tris-Glycine SDS-PAGE (Biorad) gel stained with Page-Blue (Thermo Fisher).

#### Immunoblotting and SDS-PAGE analysis

All SDS-PAGE and Western blotting experiments were performed with standard laboratory techniques^48^,^49^,^50^. The antibodies for NisB^38^, NisC^38^ and the nisin leader peptide^26^ were kindly provided by Dr. Moll, LanthioPharma, Groningen (Netherlands).

#### Visualization of the nisin modification complex by small-angle X-ray scattering (SAXS)

SAXS data were collected at ESRF Grenoble on beamline BM29 equipped with a PILATUS 1 M detector (Dectris). The sample to detector distance was kept fixed at 2.867 m. The achievable q-range under these conditions was 0.025–5 nm^−1^. The maximum measurable R_g_ (radius of gyration) of the investigated particles were 20 nm. All measurements were performed at 4 °C and the system was coupled to a size exclusion chromatography.

For analysis of the nisin modification complex, a mixture of 40 μM NisB, 320 μM NisC and 400 μM dehydrated prenisin peptide was incubated for 1 h at 25 °C. Analysis of isolated NisB was performed with a 200 μM solution and for NisC with a 470 μM solution. A volume of 200 μL was applied on a Superdex 200 10/300 increase colum (GE Healthcare) pre-equilibrated with SAXS-buffer (50 mM HEPES-NaOH, pH 7.5, 500 mM NaCl, 5% Glycerol) at a flow rate of 0.75 mL/min. For data processing we used the ATSAS Software package (Version 2.7.1)^51^. Primary data reduction were performed using the program PRIMUS^42^. The forward scattering *I*(*0*) as well as the radius of gyration (R_g_) were calculated with the Guinier approximation^52^, which is implemented in PRIMUS^42^. We calculated the pair-distribution function *p*(*r*) and estimate the maximum particle dimension (*D*_*max*_) employing the program GNOM^53^. The low resultion *ab initio* models were calculated with DAMMIF^43^ (10 runs for each sample) and used to perform the docking of the NisB-NisC dehydrated prenisin peptide complex, which was calculated with SASREF^54^. For superimposing of the models, we used SUPCOMB^44^. These programs are all part of the ATSAS program package available on the EMBL website (http://www.embl-hamburg.de/biosaxs/software.html).

## Additional Information

**How to cite this article**: Reiners, J. *et al*. Stoichiometry and structure of a lantibiotic maturation complex. *Sci. Rep.* **7**, 42163; doi: 10.1038/srep42163 (2017).

**Publisher's note:** Springer Nature remains neutral with regard to jurisdictional claims in published maps and institutional affiliations.

## Supplementary Material

Supplementary Information

## Figures and Tables

**Table 1:** MALS-SEC data summarizing the molecular weight of the complex forming analysis for the different prenisin peptide variants and without any prenisin peptide.

NisB	NisC	NisA variants	Molekular weight [kDa]
+	−	—	237.5 ± 0.3
+	−	unmodified	241.9 ± 0.4
−	+	—	48.1 ± 0.5
−	+	unmodified	54.5 ± 0.6
+	+	unmodified	293.6 ± 1.2
+	+	dehydrated	291.2 ± 0.9
+	+	modified	250.4 ± 0.7
+	+	FNLD-Box	248.0 ± 0.9

The theoretical molecular weight of a NisB dimer is calculated to 236.6 kDa, 48.5 kDa for a cleaved NisC monomer and 5.9 kDa for the unmodified prenisin peptide.
