# Supplementary material for: Stoichiometry and structure of a lantibiotic maturation complex
Source: Sci Rep. 2017 Feb 7;7:42163. doi: 10.1038/srep42163 (PMC5294574; doi:10.1038/srep42163)
Supplement: Supplementary Information [file srep42163-s1.pdf]

Supplementary material

Stoichiometry and structure of a lantibiotic maturation complex

Jens Reiners, André Abts, Rebecca Clemens, Sander H. J. Smits and Lutz Schmitt*

Institute of Biochemistry, Heinrich-Heine-University Duesseldorf, Universitaetsstraße 1, 40225 Duesseldorf, Germany

Figure S1: **Schematic overview of the nisin maturation process**. **I)** The leader peptide is shown on the top and the highly conserved -FNLD- box is highlighted by a red box. The unmodified prenisin peptide represents the ribosomally synthesized peptide. **II)** NisB dehydrates specific serine and threonine residues (grey) in the core peptide. The final peptide is called dehydrated prenisin peptide, which contains the dehydrated amino acids dehydroalanine (dha) and dehydrobutyrine (dhb). **III)** These dehydrated amino acid residues get linked via NisC to C-terminal located cysteine residues (brown), resulting in the modified prenisin peptide. In the core peptide one lanthionine ring A (orange) and four (methyl)-lanthionine rings B-E (red) are present. **IV)** After introduction of the post-translational modifications the modified prenisin peptide is secreted via NisT across the cytoplasmic membrane and processed by the serine protease NisP. The modified prenisin peptide is cleaved into the leader peptide and mature nisin. Modified from [1].

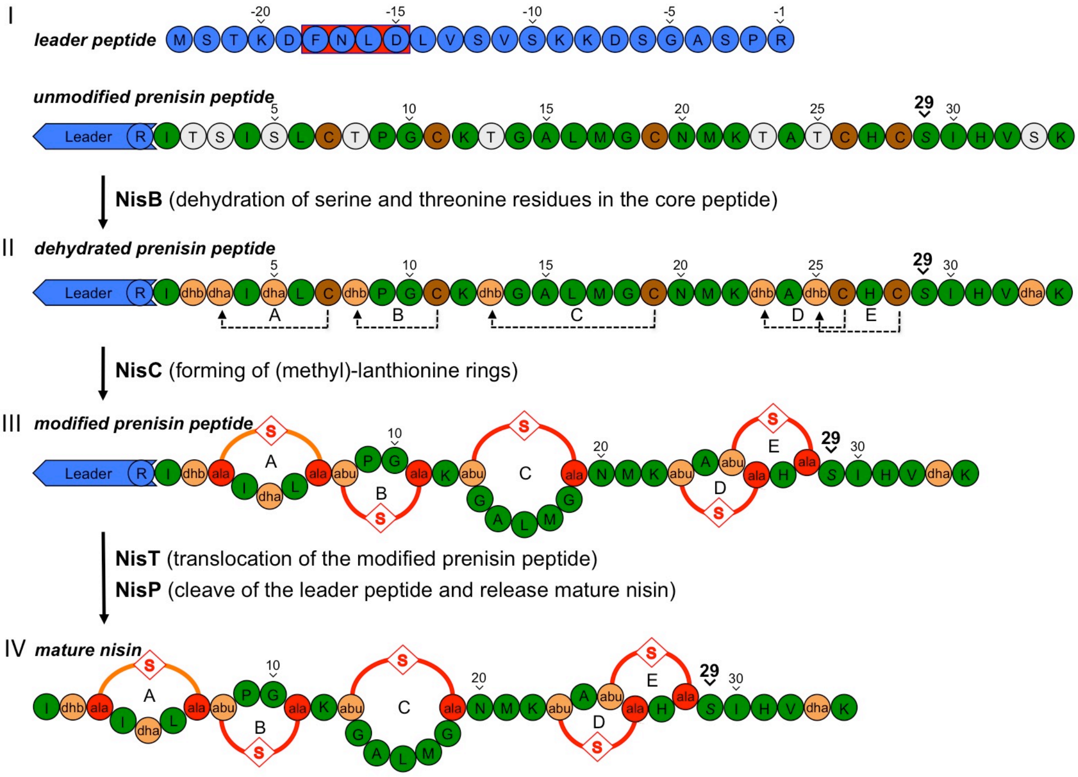

Figure S2: **SDS-PAGE analysis of the purification process of NisC and NisB, respectively. (A) NisB purification:** Lane M: protein marker. Lane 1: Crude extract of *L. lactis* cells expressing NisB-His$_6$. Lane 2: Flow through of the IMAC column. Lane 3: Combined IMAC elution fractions containing NisB-His$_6$. Lane 4: NisB-His$_6$ elution fraction after SEC. **(B) NisC purification:** Lane M: protein marker. Lane 1: Crude extract of His$_6$-NisC expressing *E. coli* cells. Lane 2: IMAC flow through. Lane 3: Combined His$_6$-NisC IMAC elution fractions. Lane 4: His$_6$-NisC elution fraction after SEC. Lane 5: Tag-free NisC after thrombin treatment. **(C)** Molecular weight determination of NisB using MALS-SEC: In black the elution profile of a purified NisB sample analyzed with MALS-SEC is shown. The observed molecular weight of 237.5 ± 0.3 kDa fits very well with the theoretical molecular weight of a NisB dimer 236.6 kDa (www.expasy.org). **(D)** The black elution profile represents the MALS-SEC analysis of a purified NisC sample. A molecular weight of 48.1 ± 0.5 kDa was determined, which is in-line with the theoretical calculated molecular weight of a cleaved NisC monomer of 48.5 kDa (www.expasy.org).

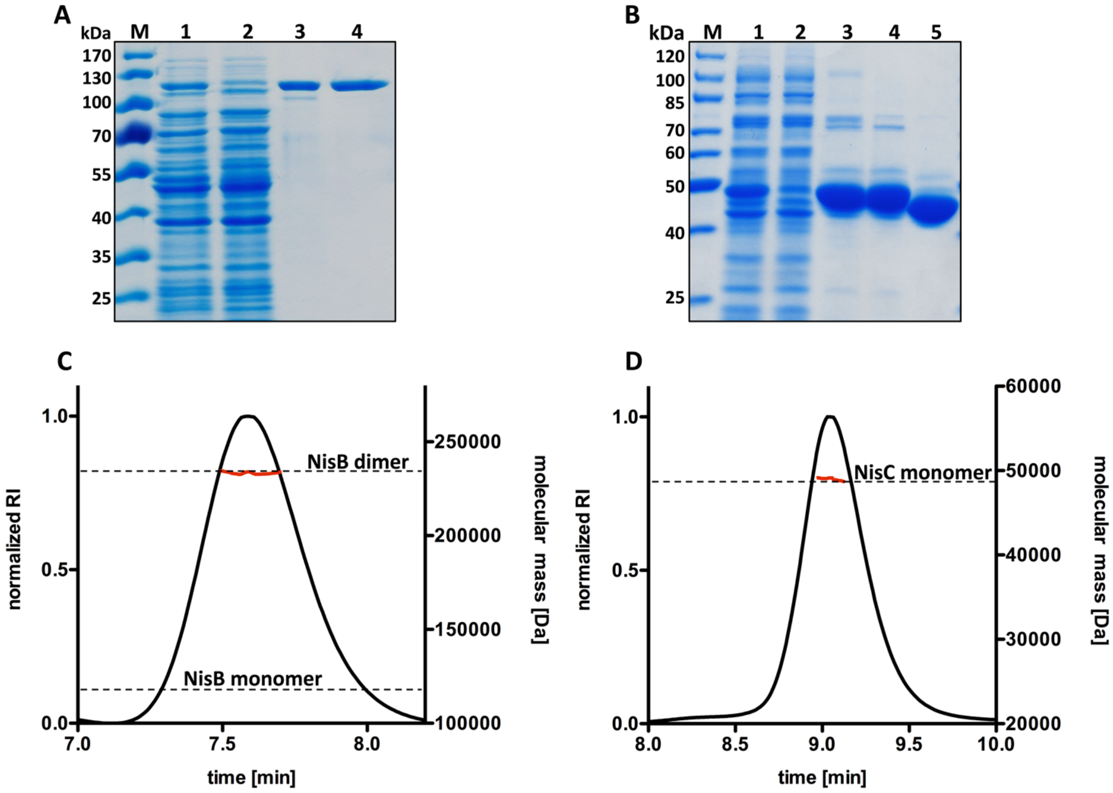

Figure S3: **Page-Blue (Thermo Fischer) stained Tricine SDS-PAGE [2] analysis of the purified prenisin peptide variants**: All variants were purified using cation exchange chromatography as previously described [1,3]. The final concentration of the purified peptide variants was determined by subjecting the prenisin peptide variants to HPLC analysis [3]. Due to space limitation several gels have been made, which are indicated by black boxes and vertical lines.

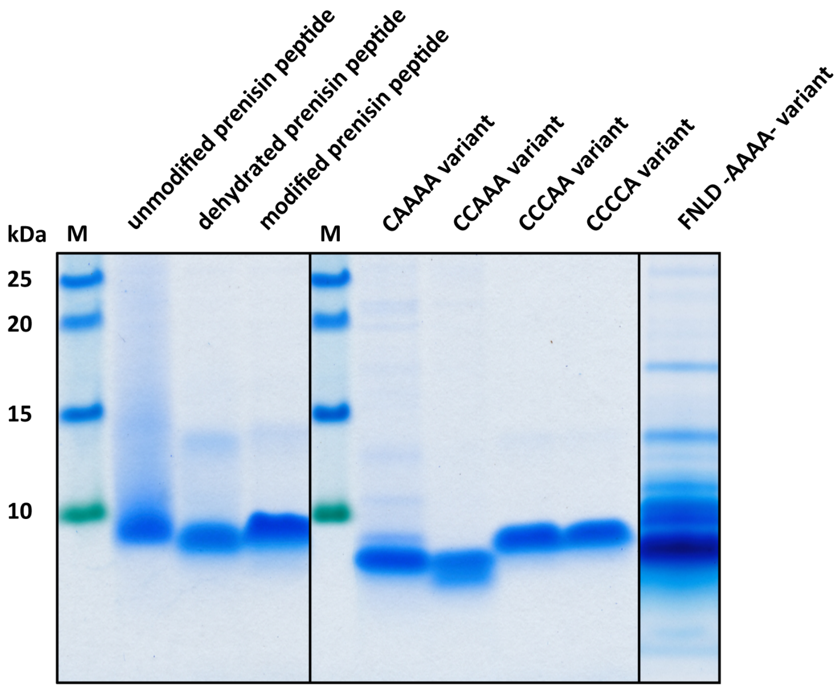

NisB and NisC substrate binding

With the MALS-SEC analysis we could show the substrate binding from NisB and NisC. The prenisin binding to NisC resulted in a changes elution time due to a change in the hydrodynamic radius (Figure S4 A, black line) in comparison to ligand-free NisC (Figure S4 A, dashed line). The observed molecular weight of ligand-free NisC was 48.1 ± 0.5 kDa. After incubation of unmodified prenisin peptide with NisC, we observed a molecular weight of 54.5 ± 0.6 kDa, which fits to NisC with one prenisin bound (theoretical molecular weight of 54.3 kDa). NisB showed no difference in the elution profile, but MALS-SEC analysis highlighted a molecular weight of 237.5 ± 0.3 kDa (Figure S4 B, blue line) for NisB and 241.9 ± 0.4 kDa for the NisB-NisA bound complex. This indicated that one unmodified prenisin peptide molecule was bound to a NisB dimer (theoretical molecular weight of 242.2 kDa). Thus, these results demonstrate a 1:1 stoichiometry for a monomer of NisC with one prenisin molecule, and also that a dimer of NisB binds one prenisin molecule. The results are in-line with previous *in vitro* studies [1,3], but in contrast to the NisB structure [4], which showed two leader peptide fragments bound within the NisB dimer.

Figure S4: **MALS-SEC analysis of the mixed protein samples consisting of NisB or NisC, in the presence and absence of prenisin peptide. (A)** The dashed black graph shows the elution profile of 20 µM NisC resulting in a molecular weight of 48.1 ± 0.5 kDa (blue line). The analysis of 20 µM NisC and 200 µM unmodified prenisin peptide is shown as the black graph, revealing an apparent molecular weight of 54.5 ± 0.6 kDa (red line) of the formed complex. **(B)** The dashed black graph represents the elution profile of 20 µM NisB resulting in a molecular weight of 237.5 ± 0.3 kDa (blue line). The analysis of 20 µM NisB and 200 µM unmodified prenisin peptide is shown as the black graph revealing an apparent molecular weight of 241.9 ± 0.4 kDa (red line) of the formed complex.

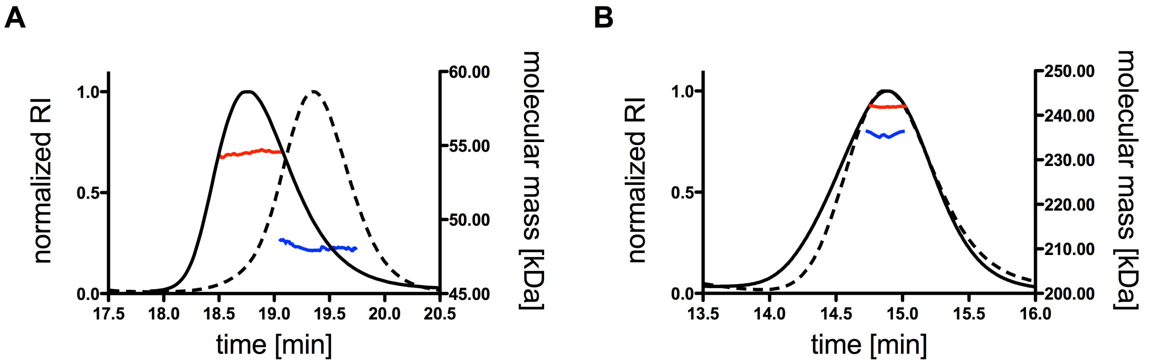

Figure S5: **SDS-PAGE analysis of fractions of size exclusion chromatograms of NisB or NisC sample as well as a NisB / NisC sample**.

The corresponding SDS gels from the size exclusion chromatography (Superdex 200 PC 3.2) fractions are shown in **(A)** 20 µM NisB (blue profile). **(B)** 160 µM NisC (red profile) **(C)** 20 µM NisB and 160 µM NisC (black profile). As observed, NisB and NisC are detected in different fractions of the SEC elution profile. The elution profiles of NisB and NisC remain unchanged in the case of the simultaneous injection of both proteins (C) indicating no complex formation. For the analysis of complex formation the fraction highlighted by a red box was used in SDS page analysis and subsequent Western blot analysis (Figure 2a).

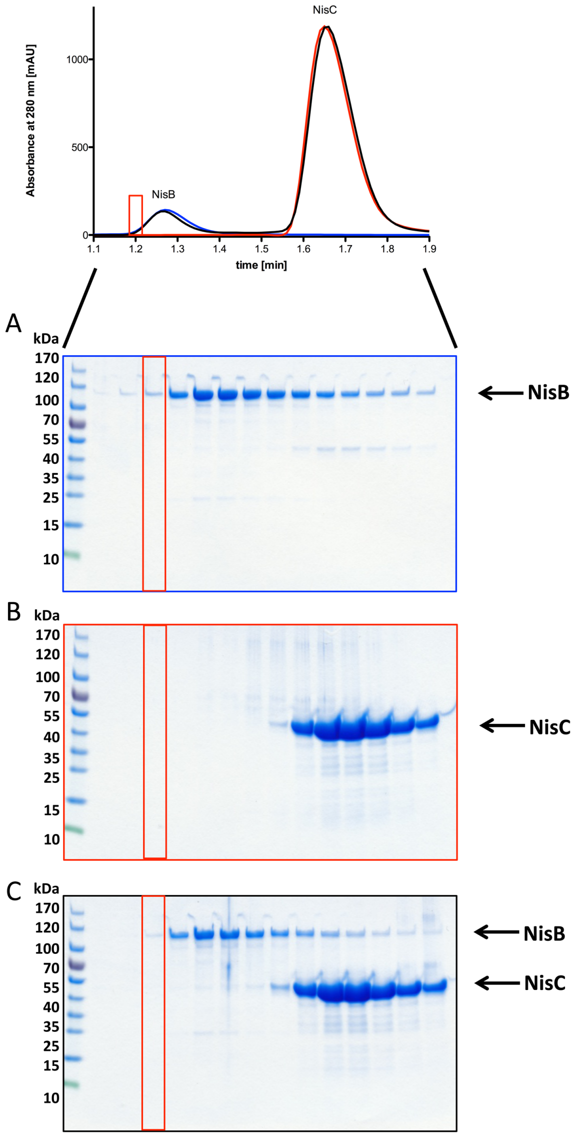

Figure S6: **MALS-SEC analysis of the pre-saturated protein samples consisting of NisB or NisC, in the presence of prenisin peptide**. **(A)** The black graph shows the elution profile of 20 µM NisB saturated with unmodified prenisin peptide and incubated with 20 µM NisC, Resulting in a molecular weight of 263.8 ± 0.3 kDa (red line). **(B)** The analysis of 20 µM NisC saturated with unmodified prenisin peptide and incubated with 20 µM NisB is shown with the black graph, revealing an apparent molecular weight of 247.1 ± 0.4 kDa (red line).

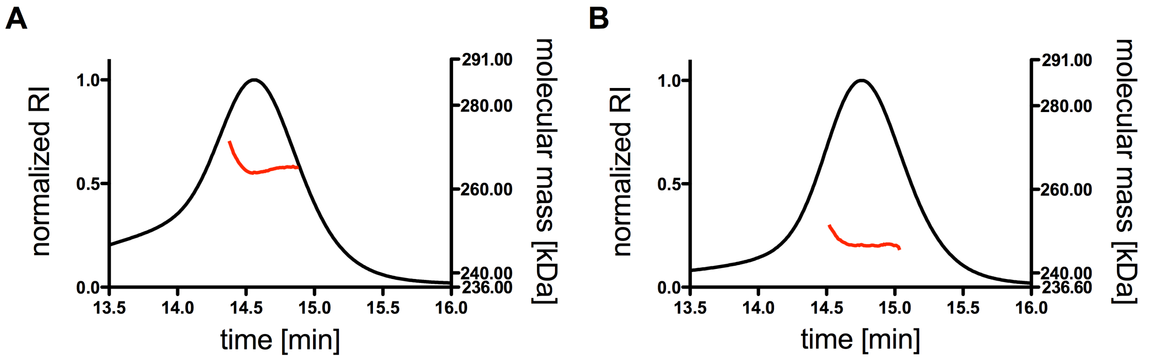

Figure S7: **SDS-PAGE analysis showing the results of the dependency of the leader and core peptides on complex formation**. The corresponding SDS gels from the size exclusion chromatography (Superdex 200 PC 3.2) fractions are shown. **(A)** 20 µM NisB and 160 µM NisC in the presence of 200 µM of the unmodified prenisin peptide. **(B)** 20 µM NisB and 160 µM NisC in the presence of 200 µM of the dehydrated prenisin peptide. **(C)** 20 µM NisB and 160 µM NisC in the presence of 200 µM of the modified prenisin peptide. **(D)** 20 µM NisB and 160 µM NisC in the presence of 200 µM of the –FNLD- box variant (-AAAA-) mutant prenisin. Red-boxed elution fractions were used for Western blot analysis (Fig. 2b-c).

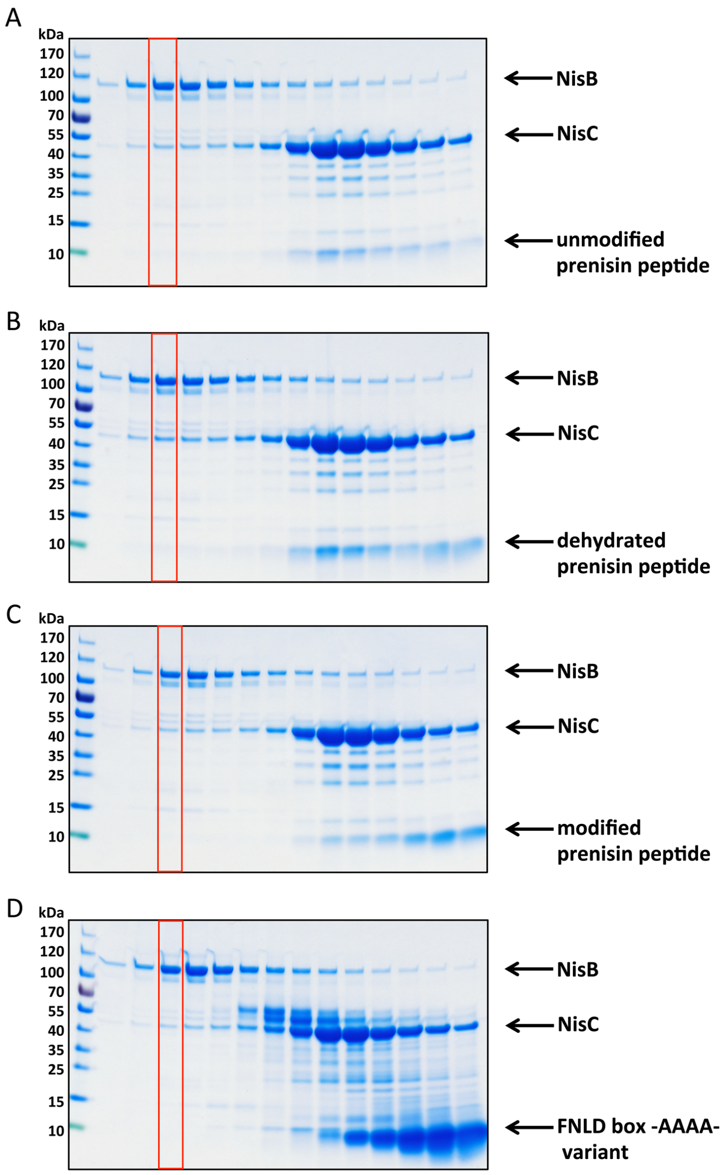

Figure S8: **SDS-PAGE analysis showing the influence in varying the number of installed (methyl)-lanthionine rings**. The corresponding SDS gels from the size exclusion chromatography (Superdex 200 PC 3.2) fractions are shown. **(A)** 20 µM NisB and 160 µM NisC in the presence of 200 µM of the CAAAA variant. **(B)** 20 µM NisB and 160 µM NisC in the presence of 200 µM of the CCAAA variant. **(C)** 20 µM NisB and 160 µM NisC in the presence of 200 µM of the CCCAA variant. **(D)** 20 µM NisB and 160 µM NisC in the presence of 200 µM of the CCCCA variant. Red-boxed elution fractions were used for the Western blot analysis (Fig. 2c).

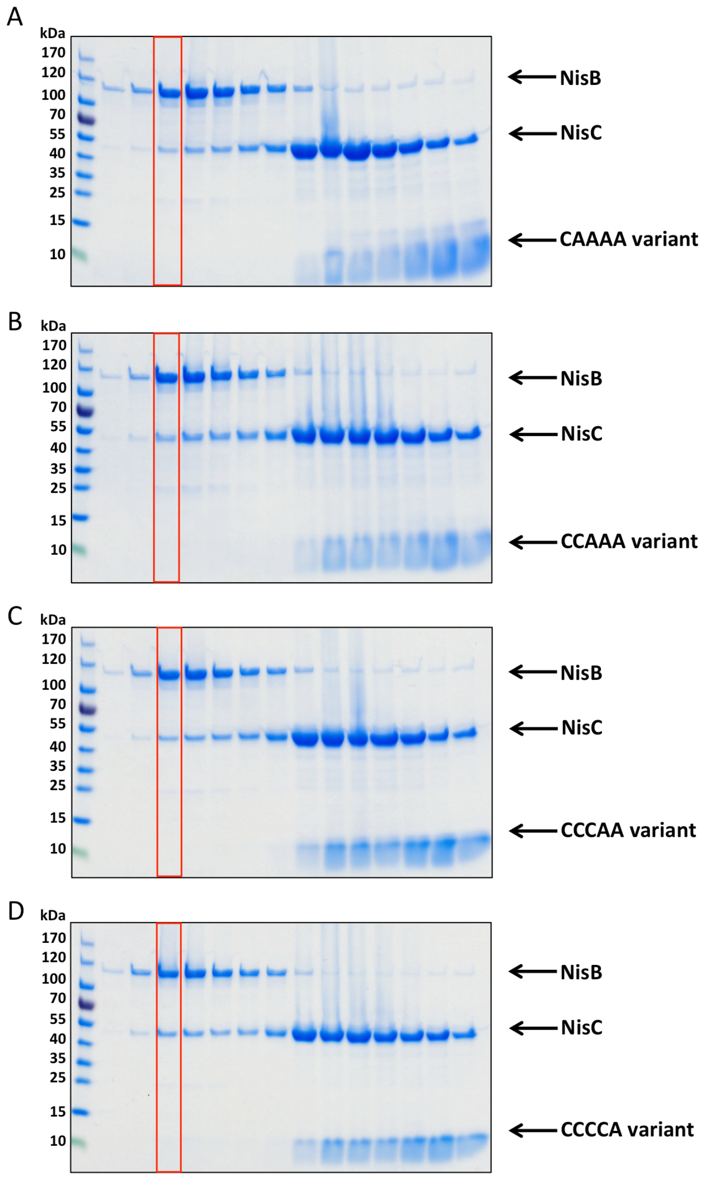

Figure S9: **SAXS plots of the different Proteins.** Experimental curves are shown in black dots and the *ab-initio* model fit as red line. Displayed is the intensity as a function of momentum transfer s. **(A)** Plot NisB, DAMMIF fit. **(B)** NisB saturated with dehydrated prenisin peptide, DAMMIF fit. **(C)** NisC, DAMMIF fit. **(D)** NisBCA complex, DAMMIF fit. **(E)** NisBCA complex, SASREF fit.

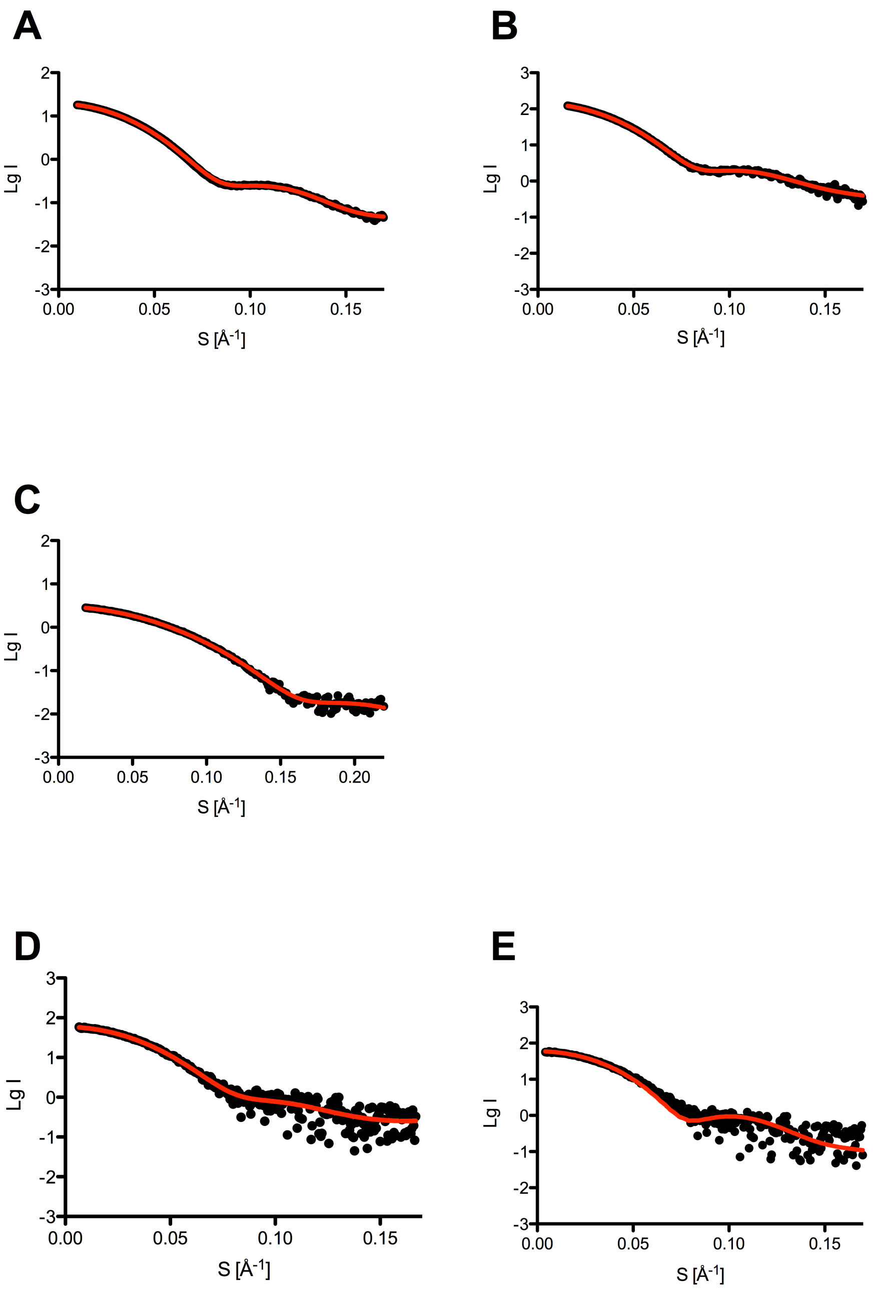

Table S1: **MALS-SEC data summarizing the molecular weight (M_w) of the analysis of complex forming for the different prenisin peptide variants.** For the analysis, we kept the concentration of NisB (20 µM) and the different prenisin peptides (200µM) constant. We only changed the concentration of NisC from 0 µM up to 160 µM. The theoretical molecular weight of a NisB dimer is calculated to 236.6 kDa, 48.5 kDa for a tag-free NisC monomer and 5.9 kDa for the unmodified prenisin peptide. All measurements were done at least in triplicate. n.d. = not determined.

**NisC concentration [µM]**	**without prenisin peptide**	**M_W nisin maturation complex [kDa]**			
		**unmodified**	**dehydrated**	**modified**	**FNLD Variant**
0	236.4 ± 0.3	n. d.	241.9 ± 0.4	n.d.	n.d.
10	n.d.	247.3 ± 1.2	249.3 ± 0.5	n.d.	n.d.
20	n.d.	256.6 ± 0.8	249.3 ± 1.1	n.d.	n.d.
40	237.5 ± 0.3	268.9 ± 1.3	260.3 ± 0.3	237.5 ± 0.8	n.d.
60	n.d.	276.8 ± 1.0	270.4 ± 0.5	n.d.	n.d.
80	238.1 ± 0.3	291.2 ± 1.1	275.5 ± 0.5	n.d.	n.d.
120	n.d.	287.2 ± 0.7	292.7 ± 0.8	n.d.	n.d.
160	238.7 ± 1.2	293.6 ± 1.2	291.2 ± 0.9	250.4 ± 0.7	248.0 ± 0.9

Table S2: **Theoretical Molecular weight from different ratios of NisB, NisC and NisA.**

The theoretical molecular weight of a NisB dimer is calculated to 236.6 kDa, 48.5 kDa for a tag-free NisC monomer and 5.9 kDa for the unmodified prenisin peptide

Ratio			
NisB	NisC	NisA	Molecular weight [kDa]
1	1	1	172.7
1	2	1	221.2
1	1	2	178.6
1	2	2	227.1
2	1	2	296.9
2	1	1	291.0
2	2	1	339.5
2	2	2	345.4

Table S3: **Overall SAXS Data**

**Data collection parameters**				
Detector	PILATUS 1 M			
Detector distance (m)	2.867			
Beam size (μm x μm)	700 x 700			
Wavelength (Å)	0.99			
Sample environment	Quartz glass capillary, 1 mm ø			
s range (nm^{-1})‡	0.025–5.0			
Temperature (°C)	4			
Exposure time per frame (s)	1.5 s continuously*			
Mode of measurement	online SEC			
**Sample**	**NisB**	**NisBA**	**NisC**	**NisBCA**
**Structural parameters**				
I(0) from P(r)	19.07	141.10	3.0	58.79
R_g (real-space from P(r)) (nm)	4.29	4.33	2.41	4.42
I(0) from Guinier fit	19.20	142.14	3.00	58.94
s-range for Guinier fit (*nm^{-1}*)	0.12 - 0.30	0.16 - 0.33	0.21 - 0.54	0.14 - 0.32
R_g (from Guinier fit, AutoR_g) (nm)	4.37	4.39	2.42	4.45
D_{max} (nm)	13.44	15.06	8.16	15.66
POROD volume estimate (nm^3)	382.82	410.47	82.5	440.17
**Molecular mass (kDa)**				
From POROD volume [kDa]	239.26	256.54	51.56	275.1
From I(0)	n.a.			
From sequence	236.6	242.4	48.5	291
**Software**				
Primary data reduction	PRIMUS [5]			
Data processing	GNOM [6]			
Ab initio modelling	DAMMIF [7]			DAMMIF[7]/SASREF[8]
Evaluation, averaging of models	DAMAVER [9]			
Superimposing	SUPCOMB [10]			
Model visualization	PyMOL			

‡s = 4πsin(θ)/λ, 2θ – scattering angle, λ – Xray-wavelength.* 0.5 s dead time between frames, n.a. not applicable.

Literature

1. Mavaro, A. et al. Substrate recognition and specificity of the NisB protein, the lantibiotic dehydratase involved in nisin biosynthesis. *J. Biol. Chem.* **286**, 30552-60 (2011).
2. Schagger, H. Tricine-SDS-PAGE. *Nat Protoc.* **1**, 16-22 (2006).
3. Abts, A., Montalban-Lopez, M., Kuipers, O.P., Smits, S.H. & Schmitt, L. NisC binds the FxLx motif of the nisin leader peptide. *Biochemistry* **52**, 5387-95 (2013).
4. Ortega, M.A. et al. Structure and mechanism of the tRNA-dependent lantibiotic dehydratase NisB. *Nature* **517**, 509-12 (2015).
5. Konarev, P.V., Volkov, V.V., Sokolova, A.V., Koch, M.H.J. & Svergun, D.I. PRIMUS: a Windows PC-based system for small-angle scattering data analysis. *J. Appl. Crystallogr.* **36**, 1277-1282 (2003).
6. Svergun, D.I. Determination of the Regularization Parameter in Indirect-Transform Methods Using Perceptual Criteria. *J. Appl. Crystallogr.* **25**, 495-503 (1992).
7. Franke, D. & Svergun, D.I. DAMMIF, a program for rapid *ab-initio* shape determination in small-angle scattering. *J. Appl. Crystallogr.* **42**, 342-346 (2009).
8. Petoukhov, M.V. & Svergun, D.I. Global rigid body modeling of macromolecular complexes against small-angle scattering data. *Biophys. J.* **89**, 1237-1250 (2005).
9. Volkov, V.V. & Svergun, D.I. Uniqueness of ab initio shape determination in small-angle scattering. *J. Appl. Crystallogr.* **36**, 860-864 (2003).
10. Kozin, M.B. & Svergun, D.I. Automated matching of high- and low-resolution structural models. *J. Appl. Crystallogr.* **34**, 33-41 (2001).
